# Clear cell adenocarcinoma of the ovary with a sarcoid reaction in the spleen and regional lymph nodes: A case report^☆^

**DOI:** 10.1016/j.gynor.2013.04.001

**Published:** 2013-04-09

**Authors:** Hiroshi Matsushima, Taisuke Mori, Tsuchiya Hiroshi, Kazuya Ooshima, Jo Kitawaki

**Affiliations:** a Department of Obstetrics and Gynecology, Kyoto Prefectural University of Medicine, Graduate School of Medical Science, 465 Kajii-cho, Kawaramachi-Hirokoji, Kamigyo-ku, Kyoto 602-8566, Japan; b Department of Obstetrics and Gynecology, Nantan General Hospital, 25 Yagiueno, Yagi-cho, Nanntann-shi, Kyoto 629-0197, Japan

**Keywords:** Epithelial ovarian cancer, Sarcoidosis, Non-caseating epithelioid cell granuloma

## Abstract

• Preoperative diagnosis of sarcoid reactions is important to avoid overtreatments.
• Sarcoid reactions could act as a favorable prognostic factor in ovarian cancer.

## Introduction

A sarcoid reaction is defined as a phenomenon representing histologically proven granulomatous lesions without evidence of sarcoidosis. Although several reports have been published on sarcoid reactions accompanying malignant tumors (Herxheimer, 1917; Brincker, 1986), to our knowledge, no study to date has shown an association between sarcoid reactions and epithelial ovarian cancer. In this report, we present a case of clear cell adenocarcinoma of the ovary co-existing with a sarcoid reaction in the lymph nodes and spleen.

## Case report

A 68-year-old Japanese woman, gravida 2 para 2 (G_2_P_2_), was referred to our institution with a complaint of irregular genital bleeding. Her medical and family history was unremarkable. The last gynecologic examination and cervical smear had been performed 3 years previously, and the findings were normal.

**Fig. 1:**
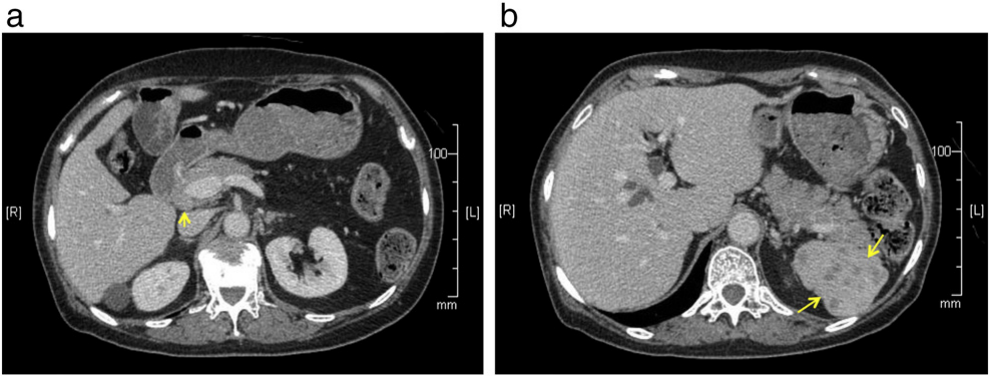
(a) CT scan showing swellings in the lymph nodes (arrows). (b) Low-density areas in the spleen were also observed (arrows), suggesting metastasis from a malignant tumor of the left ovary.

**Fig. 2:**
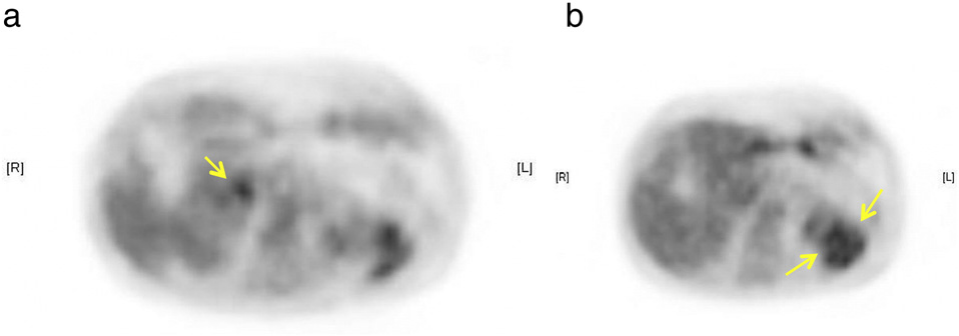
(a) ^18^FDG avidity was observed in the lymph nodes (arrows) and (b) in the spleen (arrows).

On physical examination, we observed a palpable goose-egg sized tumor in the lower abdomen. Magnetic resonance imaging revealed a hard, abnormal cystic tumor mass in the pelvis, 100 × 103 mm in size, suggesting a malignant ovarian tumor. Computed tomography (CT) of the lower abdomen revealed bulging lymph node swellings, each 10 mm in size, along the bilateral common iliac arteries with lesions to both the external and internal arteries. The CT scan also revealed a > 10-mm lymph node swelling in the dorsal pancreas (Fig. 1a) and multiple low-density areas in the spleen (Fig. 1b). These findings suggested metastasis from a primary ovarian cancer. Following whole body ^18^F-fluorodeoxyglucose positron emission tomography (FDG-PET), elevated FDG uptake was reported in the left adnexa, in the lymph nodes along the iliac arteries, in the dorsal pancreas and spleen (Fig. 2a,b).

With evidence of malignancy originating from the left ovary and subsequent multiple lymph node metastases and metastasis to the spleen, we performed abdominal hysterectomy, bilateral salpingo-oophorectomy, pelvic para-aortic lymphadenectomy, and splenectomy. During surgery, no remarkable dissemination was detected in the abdominal cavity. As observed on CT and FDG-PET, enlarged lymph nodes were visible around both bilateral common iliac and external/internal iliac lesions as well as around the pancreatic lesion, and these were all resected.

**Fig. 3:**
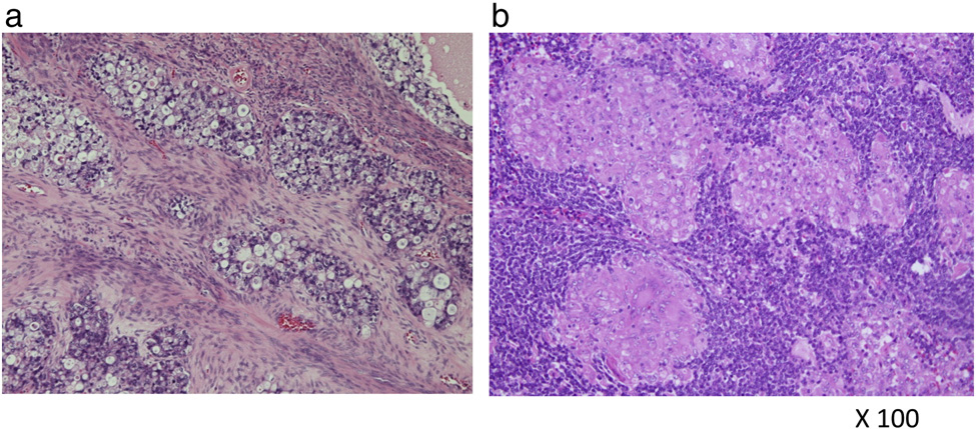
(a) Microscopic findings of the resected ovarian tumor and lymph nodes. Atypical cells with clear cytoplasm grew papillary, tubulocystic, and focally solid pattern (hematoxylin and eosin [HE]). (b) Non-caseating epithelioid granulomas were observed in the pelvic lymph node as well as in the spleen where there were no metastatic lesions (HE).

Histopathological findings showed the growth of tumor cells in papillary, tubulocystic, and focally solid pattern composed of cells with clear cytoplasm, hyperchromatic nuclei and mitotic features. Specifically, a distinct hobnail pattern was observed (Fig. 3a). No tumor cells were recorded on the right side of the ovary. Histopathological examination of the resected lymph nodes and spleen revealed a non-caseating epithelioid cell granuloma (Fig. 3b), wherein no tumor cells were identified. On the basis of these findings, we concluded that this was a case of clear cell adenocarcinoma of the left ovary, p-T1aN0M0. The patient received adjuvant chemotherapy with paclitaxel [180 mg/m^2^] and carboplatin [AUC 5], q3 weeks × 6 courses. After 2 years of follow-up, no recurrence of disease was noted.

## Discussion

Since the first study by Herxheimer (1917), several reports have been published on sarcoid reactions associated with malignant tumors. Brincker et al. (1986) reported that 4.4% of solid tumors co-exist with sarcoid reactions, many of which correlate with carcinoma rather than sarcoma and, histologically, are detected in squamous cell carcinoma rather than in adenocarcinoma. Although several studies in the literature report the association of sarcoid reactions with other kinds of primary organs, including the stomach, lung, and liver, no report to date has shown a link to epithelial ovarian cancer.

The cause of sarcoid reactions associated with malignant tumors remains controversial for a variety of reasons (e.g., local non-specific reaction to tumor cells, tissue reaction to tumor embolism in the lymphatic and blood vessels, mucosal injury, abnormal local immune response, or autoimmune reaction caused by tumor-derived soluble antigen) (Kojima et al., 1997; Neiman, 1977). It has been suggested that T-cell-mediated immune response is associated with the pathophysiology of sarcoidosis. Additionally many reports recently have hypothesized that dendritic cells play an important role in the mechanism of T-cell activation that leads to formation of granulomas (Ota et al., 2004). Kurata et al. (2005) reported that, also in a sarcoid reaction, the immune response caused by T-cell activation of dendritic cells contributed to granuloma formation. In our study, following immunostaining for dendritic cell markers S100, we confirmed the existence of mature dendritic cells in the granulomas (data not shown).

Furthermore, patients with Hodgkin's disease or gastric cancer, who display sarcoid reaction, have been reported to exhibit better prognosis than those with no observable sarcoid reaction (Kurata et al., 2005; Sacks et al., 1978). In addition, Zhang et al. (2003) reported that T-cell invasion into the tumor could improve prognosis in advanced ovarian carcinoma. Thus, sarcoid reaction caused by T cell-mediated autoimmune reaction may be considered a factor for improved prognosis in patients with ovarian cancer, as well as those with Hodgkin's disease or gastric cancer. Herein, we have described a case where no lymph node metastasis was detected and no recurrence of disease was observed.

We routinely use ^18^F-FDG-PET to evaluate the preoperative staging because previous reports demonstrated that PET/contrast-enhanced CT could be more accurate and useful modality for surgical decision making of ovarian cancer (Kitajima et al., 2008). However, it is extremely difficult to differentiate between malignant and sarcoid lesions by using ^18^F-FDG-PET because sarcoid lesions are ^18^F-FDG avid. In this patient, we concluded that metastasis from a left ovary primary cancer was present on the basis of the ^18^F-FDG-PET results. Therefore, we performed histopathological testing following the lymphadenectomy and splenectomy. It has been shown that ^18^F-α-methyl tyrosine (FMT)-PET, used for the imaging of amino acid metabolism, shows a higher specificity for malignant disorder than did FDG-PET, and thus the former could be used to clearly differentiate between granulomatous and metastatic lesions (Pavic et al., 2008). As a result, we propose the introduction of this technique in future clinical practice. But, eventually, histopathological analysis is indispensable to confirm whether the lesion is benign or malignant. Therefore, intra-operative frozen sections should be performed, when the possibility of sarcoid reaction was suggested.

In conclusion, sarcoid reaction is a phenomenon which can occur in any kind of malignant diseases, so when multiple metastasis is suspected, the possibility of sarcoid reaction should be taken into account. Furthermore it is important to first clarify the mechanism underlying sarcoid reaction in order to establish the preoperative diagnosis, thereby reducing the need for expanded surgery and its associated complications with the aim of optimizing the patient's quality of life.

## Conflict of interest statement

The authors declare that there are no conflicts of interest.
